# miR-632 Induces DNAJB6 Inhibition Stimulating Endothelial-to-Mesenchymal Transition and Fibrosis in Marfan Syndrome Aortopathy

**DOI:** 10.3390/ijms242015133

**Published:** 2023-10-13

**Authors:** Sonia Terriaca, Maria Giovanna Scioli, Calogera Pisano, Giovanni Ruvolo, Amedeo Ferlosio, Augusto Orlandi

**Affiliations:** 1 Anatomic Pathology, Department of Biomedicine and Prevention, Tor Vergata University, 00133 Rome, Italy; terriacasonia093@gmail.com (S.T.); ferlosio@med.uniroma2.it (A.F.); orlandi@uniroma2.it (A.O.); 2 Cardiac Surgery, Department of Surgical Sciences, Tor Vergata University, 00133 Rome, Italy; lindapisano82@gmail.com (C.P.); giovanni.ruvolo@uniroma2.it (G.R.)

**Keywords:** Marfan syndrome, thoracic aortic aneurysms, aortic wall degeneration, endothelial-to-mesenchymal transition, fibrosis, TGF-β1, miR-632, DNAJB6

## Abstract

Marfan syndrome (MFS) is a connective tissue disorder caused by *FBN1* gene mutations leading to TGF-β signaling hyperactivation, vascular wall weakness, and thoracic aortic aneurysms (TAAs). The pathogenetic mechanisms are not completely understood and patients undergo early vascular surgery to prevent TAA ruptures. We previously reported miR-632 upregulation in MFS TAA tissues compared with non-genetic TAA tissues. DNAJB6 is a gene target of miR-632 in cancer and plays a critical role in blocking epithelial-to-mesenchymal transition by inhibiting the Wnt/β catenin pathway. TGF-β signaling also activates Wnt/β catenin signaling and induces endothelial-to-mesenchymal transition (End-Mt) and fibrosis. We documented that miR-632 upregulation correlated with DNAJB6 expression in both the endothelium and the tunica media of MFS TAA (*p* < 0.01). Wnt/β catenin signaling, End-Mt, and fibrosis markers were also upregulated in MFS TAA tissues (*p* < 0.05, *p* < 0.01 and *p* < 0.001). Moreover, miR-632 overexpression inhibited *DNAJB6*, inducing Wnt/β catenin signaling, as well as End-Mt and fibrosis exacerbation (*p* < 0.05 and *p* < 0.01). TGF-β1 treatment also determined miR-632 upregulation (*p* < 0.01 and *p* < 0.001), with the consequent activation of the aforementioned processes. Our study provides new insights about the pathogenetic mechanisms in MFS aortopathy. Moreover, the high disease specificity of miR-632 and DNAJB6 suggests new potential prognostic factors and/or therapeutic targets in the progression of MFS aortopathy.

## 1. Introduction

Marfan syndrome (MFS) is a connective tissue disorder caused by mutations in the *FBN1* gene, which codes for the extracellular glycoprotein fibrillin-1 [1]. Approximately 75% of MFS patients have affected relatives, while the remaining 25% carry de novo mutations in the FBN1 gene [2]. The diagnosis of MFS can be complex, due to both a phenotypic variability in the MFS population and an overlap of Marfan’s manifestations with other connective-tissue diseases. Moreover, some of those manifestations depend on age [2]. An estimated prevalence of MFS is approximately 1 case per 3000 to 5000 individuals, regardless of sex [2]. Fibrillins interact with different components of the extracellular matrix (ECM), such as elastin, collagen, fibronectin, and vitronectin [3]. *FBN1* mutations in the aorta cause the formation of loose and disordered elastic fibers, leading to a weakened vascular wall and the consequent early formation of thoracic aortic aneurysms (TAAs) [2].

Moreover, fibrillin domains, through the latent transforming growth factor β binding proteins (LTBP), are also involved in the sequestration of latent TGF-β [4]. Therefore, fibrillin-1 alterations lead to an impaired sequestration of latent TGF-β, resulting in increased free TGF-β [5]. As reported above, despite multiple clinical features, the most life-threatening clinical manifestation in MFS patients concerns the cardiovascular system. In particular, the early development and rapid progression of untreated TAAs may lead to aortic wall dissection and/or rupture [6]. From a clinical–pathological point of view, TAAs in MFS patients show evident similarities to non-genetic TAAs [7]. Currently, the pharmacological treatments available for MFS patients are antihypertensive drugs, such as β-blockers, which counteract the excessive activation of TGF-β signaling [8]. When pharmacological treatments are not effective, patients undergo surgery to remove the portions of the aorta that gradually but rapidly tend to dilate [9,10].

In the last few years, the study of gene regulation in different cardiovascular diseases brought to light the presence of important alterations in the microRNAs (miRNAs) regulatory system [11,12]. miRNAs are small non-coding RNAs that regulate gene expression post-transcriptionally. miRNAs generally interact with the 3′ untranslated region (3′-UTR) of target mRNAs, thereby inducing their degradation and translational silencing [13]. The deregulation of miRNA expression has been associated with many human diseases, including MFS [13,14]. Recently, Welten et al. discussed the critical role of specific miRNAs in vascular remodeling and in TAA development [15]. In addition, deregulation of specific miRNAs in the peripheral blood of MFS patients could represent a potential prognostic tool for cardiovascular complications [13]. We previously demonstrated a significant upregulation of miR-632 in MFS TAAs [7]. Bioinformatics analysis (mirPath v.3—DIANA TOOLS) correlated miR-632 with the TGF-β pathway. 

In the relevant literature, it has been reported that miR-632 targets the human DnaJ/Hsp40 family member B6 (DNAJB6) in breast cancer [16]. DNAJB6 is a constitutively expressed member of the DNAJ family, the members of which are characterized by a highly conserved amino acid trait called “J domain”. The latter belongs to one of two major classes of molecular chaperones that are involved in cellular events, such as protein folding and the complex assembly of oligomeric proteins [17]. In metastatic breast cancer, DNAJB6 downregulation correlated with miR-632 upregulation [16]. Moreover, DNAJB6 has been suggested to have a protective role against the epithelial-to-mesenchymal transition (Emt) in breast cancer [18]. In particular, DNAJB6 activates the Dickkopf-1 (DKK1) factor, which in turn blocks LRP5/6 (the Wnt coreceptor) and, consequently, the Wnt/β catenin signaling that is involved in Emt and the metastatic process [19]. It has been reported that TGFβ/Wnt-β catenin signaling is involved in endothelial-to-mesenchymal transition (End-Mt) [20], a process that is also observed in the aortic aneurysms [21]. Moreover, the TGFβ/Wnt-β catenin signaling is reported to be involved in the fibrotic process [22,23]. In this study, we investigated the involvement of miR-632 and its gene target DNAJB6 in TGFβ/Wnt-β catenin signaling, End-Mt, and the fibrotic process in order to deepen the understanding of the pathogenetic role of miR-632 in MFS aortopathy.

## 2. Results

### 2.1. miR-632 Upregulation Associates with DNAJB6 Inhibition in MFS TAAs

**Figure 1:**
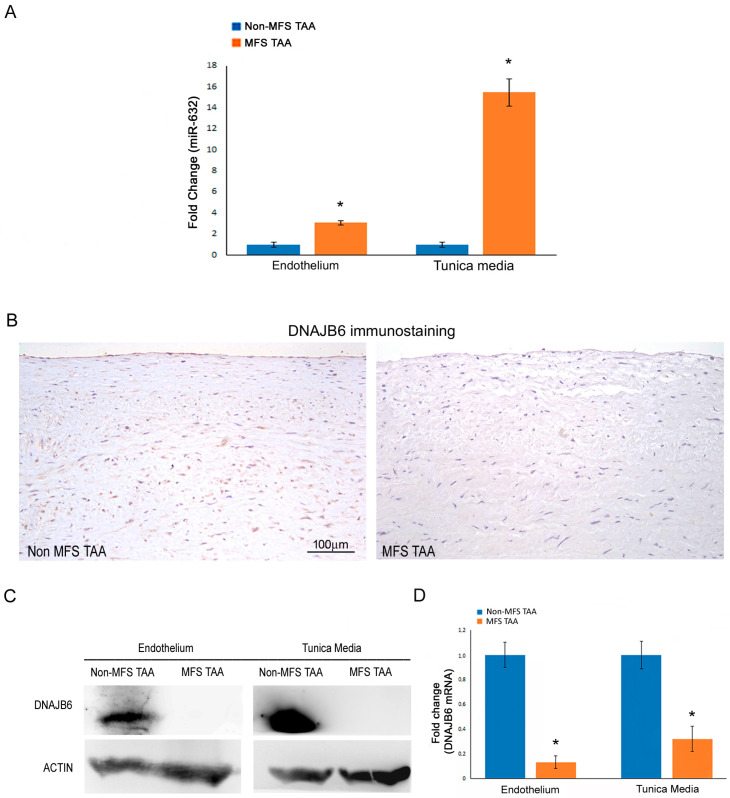
miR-632 upregulation associates with DNAJB6 inhibition in MFS TAAs. (**A**) Gene expression analysis shows a greater upregulation of miR-632 in the endothelium and the tunica media of MFS compared with non-MFS TAA. (**B**) Representative images of DNAJB6 immunostaining document the almost complete absence of the protein in MFS TAA. Instead, DNAJB6 expression is evident in the endothelium and the tunica media of non-MFS TAA. Scale bar = 100 µm. (**C**) Representative blots display an undetectable signal for DNAJB6 in the endothelium and the tunica media of MFS TAA. (**D**) Gene expression analysis shows a significant downregulation of *DNAJB6* in the endothelium and the tunica media of MFS TAA. The results are reported as the mean ± SEM. Immunohistochemical analysis was performed on MFS TAA tissue samples (*n* = 30) and non-MFS TAA tissue samples (*n* = 30). Biomolecular analyses were carried out on two different pooled samples (two for non-MFS and two for MFS; five samples for each pool), totaling ten MFS TAA patients and ten non-MFS TAA patients. Unpaired *t*-test: * indicates *p* < 0.01; 4 < Cohens’ d < 11.

In order to deepen our understanding of the regulatory role of miR-632, we performed gene expression analyses on endothelial and medial pools from MFS and non-MFS TAA tissue samples. We confirmed the upregulation of miR-632 in the MFS TAA tunica media and documented, for the first time, this deregulation in the endothelium as well (Figure 1A; *p* < 0.01; d = 10.77 and d = 5.99).

Successively, we checked the modulation of DNAJB6 in aortic samples—in particular, in endothelial and medial pools derived from MFS and non-MFS TAA patients. An immunohistochemical evaluation of tissue slides (Figure 1B) showed DNAJB6 expression almost exclusively in the tunica media and endothelium of non-MFS TAAs, in both the nucleus and cytoplasm. Blot analysis revealed an undetectable signal for DNAJB6 protein (in our lab system) in the endothelium and the tunica media of MFS TAA samples (Figure 1C). Finally, gene expression analysis showed a stronger *DNAJB6* downregulation in MFS TAA samples than in non-MFS TAA samples (Figure 1D; *p* < 0.01; d = 4.44 and d = 6.21). 

### 2.2. A Marked Endothelial-Mesenchymal Transition Characterizes MFS TAA Endothelium 

**Figure 2:**
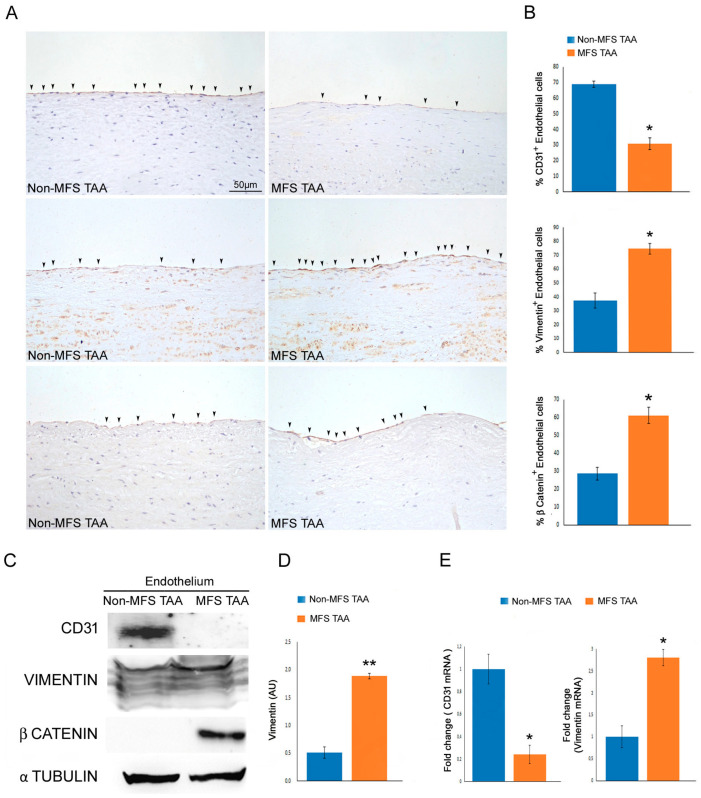
Endothelial-to-mesenchymal transition is strongly accentuated in MFS TAAs. (**A**) Representative images and (**B**) semiquantitative evaluation of CD31, vimentin, and β catenin immunostainings display a reduced percentage of CD31^+^ cells (arrow heads), as well as an increased percentage of vimentin (arrow heads) and β catenin (arrow heads) in the endothelium of MFS TAA compared with non-MFS TAA. The results are reported as the mean ± SEM. Scale bar = 50 µm. (**C**) Representative blots and (**D**) densitometric analysis show a significant downregulation of CD31, as well as an upregulation of vimentin and β catenin in the endothelium of MFS TAA compared with non-MFS TAA. (**E**) Gene expression analysis documents the transcript levels of *CD31* and *VIMENTIN* in non-MFS TAA and MFS TAA endothelium. Immunohistochemical analysis was performed on MFS TAA tissue samples (*n* = 30) and non-MFS TAA tissue samples (*n* = 30). Biomolecular analyses were carried out on two different pooled samples (two for non-MFS and two for MFS; five samples for each pool), totaling ten MFS TAA patients and ten non-MFS TAA patients. Unpaired *T*-test: * and ** indicate *p* < 0.05 and *p* < 0.01, respectively; 1 < Cohen’s d < 7.

End-Mt has been described in endothelial cells of exacerbated aortic aneurysms [21]. In this study, we compared the expression of endothelial and mesenchymal markers in MFS TAA samples and non-MFS TAA samples. Immunohistochemistry showed a reduced percentage of CD31^+^ endothelial cells, as well as a higher percentage of vimentin^+^ and β catenin^+^ endothelial cells in the MFS TAA samples (Figure 2A,B; *p* < 0.05: d = 2.30, d = 1.52 and d = 1.46). Western blotting confirmed those immunohistochemical results, displaying a strong accumulation of β catenin and vimentin, as well as an almost undetectable CD31 expression in MFS TAA endothelium compared with non-MFS TAA endothelium (Figure 2C,D; *p* < 0.01; d = 6.09). Gene expression analysis also confirmed *CD31* downregulation and *vimentin* upregulation in MFS TAA endothelium (Figure 2E; *p* < 0.05: d = 5.05 and d = 4.52). Those data evidenced a more pronounced mesenchymal phenotype in the endothelial cells of MFS TAA.

### 2.3. MFS TAA Is Characterized by an Increased Dedifferentiation of Medial Smooth Muscle Cells and Fibrosis

**Figure 3:**
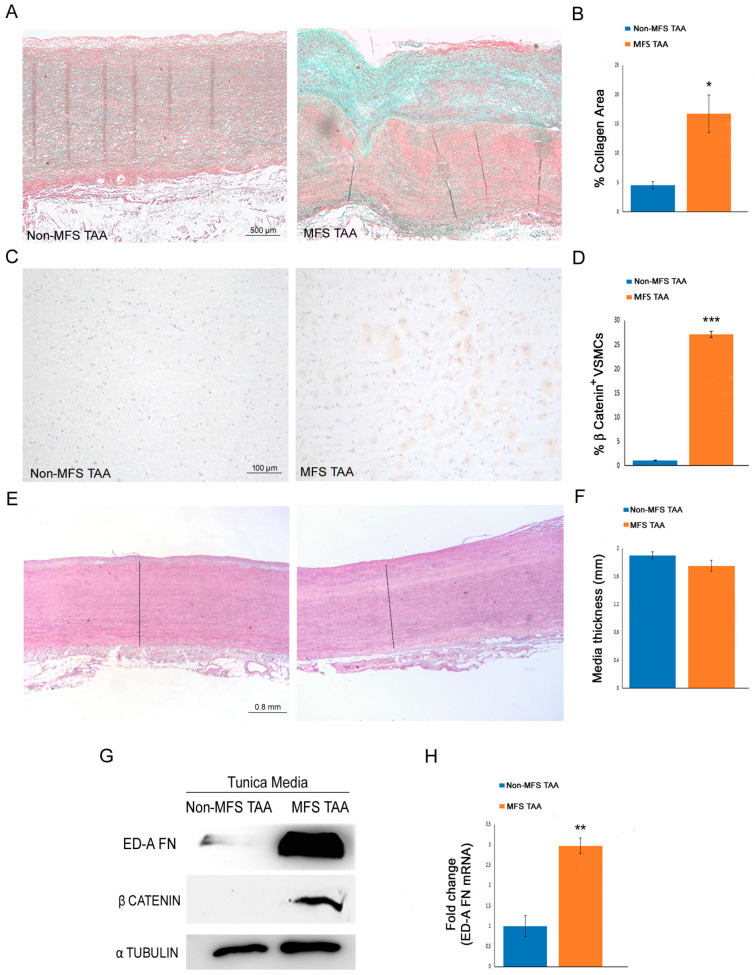
A marked fibrosis is present in the tunica media of MFS TAAs. (**A**) Representative images and (**B**) morphometric analysis of Masson’s trichrome-stained tunica media sections display a higher percentage of collagen area in MFS TAA than in non-MFS TAA. Scale bar = 500 µm. The results are reported as the average percentage of the collagen areas ± SEM. (**C**) Representative images and (**D**) immunohistochemistry evaluation of β catenin show an increased expression in MFS TAA compared with non-MFS TAA. Scale bar = 100 µm. The results are reported as the average percentage of the positive cells/field (at 20× magnification) ± SEM. (**E**) Representative images of hematoxylin and eosin-stained aorta tissue sections and (**F**) morphometric evaluation of medial thickness document similar values for non-MFS TAA and MFS TAA. Scale bar = 0.8 mm. (**G**) Representative blots for ED-A FN and β catenin display an increased protein expression in MFS TAA media. (**H**) Gene expression analysis shows a significant upregulation of *ED-A FN* transcripts in the tunica media of MFS TAA compared with non-MFS TAA. The results are reported as the mean ± SEM. Histochemical and immunohistochemical analyses were performed on MFS TAA tissue samples (*n* = 30) and non-MFS TAA tissue samples (*n* = 30). Biomolecular analyses were carried out on two different pooled samples (two for non-MFS and two for MFS; five samples from each pool), totaling ten MFS TAA patients and ten non-MFS TAA patients. Unpaired *t*-test: *, ** and *** indicate *p* < 0.05, *p* < 0.01 and *p* < 0.001, respectively; 2 < Cohen’s d < 11.

The upregulation of the Wnt/β catenin signaling is reported to be associated with dedifferentiation of smooth muscle cells toward a myofibroblast phenotype with increased fibrotic activity [24]. Masson’s trichrome staining of MFS TAA tissues and non-MFS TAA tissues documented that the percentage of fibrotic area, as an inhomogeneous deposition of collagen, was remarkably higher in the tunica media of MFS TAA than in that of non-MFS TAA (Figure 3A,B; *p* < 0.05;d = 3.06). Nevertheless, the medial thickness was similar between non-MFS TAAs and MFS TAAs (Figure 3E,F). Moreover, we documented that β catenin expression was strongly upregulated (Figure 3C,D,G; *p* < 0,001; d = 10.19). We also reported an increased expression of the domain A of fibronectin (ED-A FN), a marker of fibrosis [25], in the tunica media of MFS TAA (Figure 3G,H; *p* < 0.01; d = 2.20). 

### 2.4. miR-632 Overexpression Induces a Strong Inhibition of DNAJB6 

**Figure 4:**
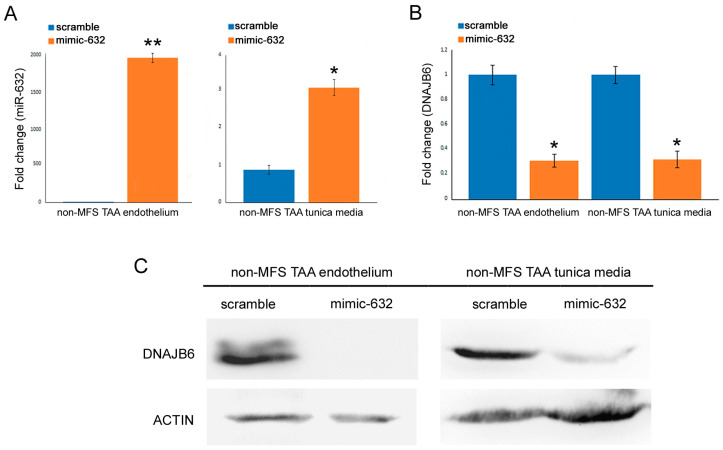
miR-632 overexpression inhibits DNAJB6 levels. (**A**) Gene expression analysis confirms the overexpression of miR632 in the endothelium and the tunica media of non-MFS TAA transfected with miR-632 mimic (50 µm) at 24 h. (**B**) *DNAJB6* is downregulated in the endothelium and the tunica media of miR-632 mimic-transfected non-MFS TAA. The results are reported as the mean ± SEM. (**C**) Representative blots confirm the inhibition of DNAJB6 in the endothelium and the tunica media of miR-632 mimic-transfected non-MFS TAA. Experiments were performed in triplicate on one sample at a time for a total of six patients collected in two different pools (two for scramble and two for mimic-632; three samples for each pool). Unpaired *t*-test: * and ** indicate *p* < 0.01 and *p* < 0.001, respectively; 4 < Cohen’s d < 12.

In order to confirm DNAJB6 as a target of miR-632, we transfected aortic fragments of non-MFS TAA tissue, in which miR-632 was poorly expressed, with an miR-632 mimic. As shown in Figure 4A, miR-632 expression was upregulated in the endothelium and the tunica media of the non-MFS TAA samples after 24 h transfection (Figure 4A; *p* < 0.001 and *p* < 0.01; d = 11.75 and d = 6.86). The overexpression of miR-632 induced a significant downregulation of DNAJB6 expression in both the endothelium and the tunica media (Figure 4B,C; *p* < 0.01; d = 6.25 and d = 4.29). 

### 2.5. miR-632 Overexpression Exacerbates Endothelial-to-Mesenchymal Transition and Smooth Muscle Cell Dedifferentiation

**Figure 5:**
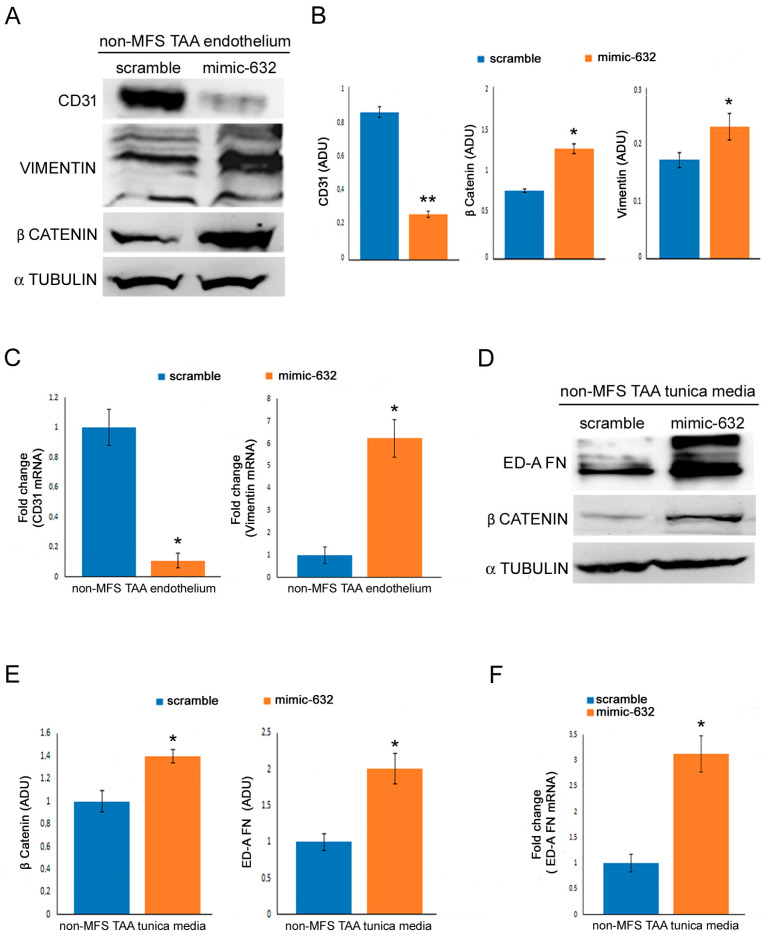
miR-632 overexpression increased endothelial-to-mesenchymal transition and fibrosis. (**A**) Representative blots and (**B**) densitometric analysis show a reduced expression of CD31 and increased levels of vimentin and β catenin in the endothelium of miR-632 mimic-transfected non-MFS TAA. (**C**) Gene expression analysis documents a significant downregulation of *CD31* and upregulation of *VIMENTIN* in the endothelium of miR-632 mimic-transfected non-MFS TAA. (**D**) Representative blots and (**E**) densitometric analysis display an increased expression of ED-A FN and β catenin in the tunica media of miR-632 mimic-transfected non-MFS TAA. (**F**) Gene expression analysis confirms a significant upregulation of *ED-A FN* in the tunica media of miR-632 mimic-transfected non-MFS TAA. The results are reported as the mean ± SEM. Experiments were performed in triplicate on one sample at a time for a total of six patients collected in two different pools (two for scramble and two for mimic-632; three samples for each pool). Unpaired *t*-test: * and ** indicate *p* < 0.05 and *p* < 0.01, respectively; 2 < Cohen’s d < 11.

We investigated if DNAJB6 downregulation, induced by miR-632 overexpression, was responsible for the increased End-Mt observed in MFS TAA. As shown in Figure 5, the CD31 marker was downregulated, while β catenin and vimentin were upregulated in the endothelium of mimic-632-transfected non-MFS TAA tissue fragments (Figure 5A,B; *p* < 0.01 and *p* < 0.05; d = 10.84, d = 9.5 and d = 9.6; Figure 5C; *p* < 0.05; d = 4.16 and d = 3.35), suggesting a regulatory role of miR-632 in the End-Mt of endothelial cells. The deregulation of Wnt/β catenin signaling has been reported to be involved in myofibroblast differentiation and fibrosis [26]. Therefore, we analyzed the effects of miR-632 overexpression on the Wnt/β catenin signaling in the tunica media of miR-632 mimic-transfected non-MFS TAA. Gene expression analysis and blots demonstrated that miR-632 overexpression induced β catenin accumulation and ED-A FN expression (Figure 5D,E; *p* < 0.05; d = 9.84 and d = 4.65; Figure 5F *p* < 0.05; d = 2.52), suggesting a crucial role of miR-632 in the dedifferentiation of smooth muscle cell phenotypes and their switch in myofibroblasts. 

**Figure 6:**
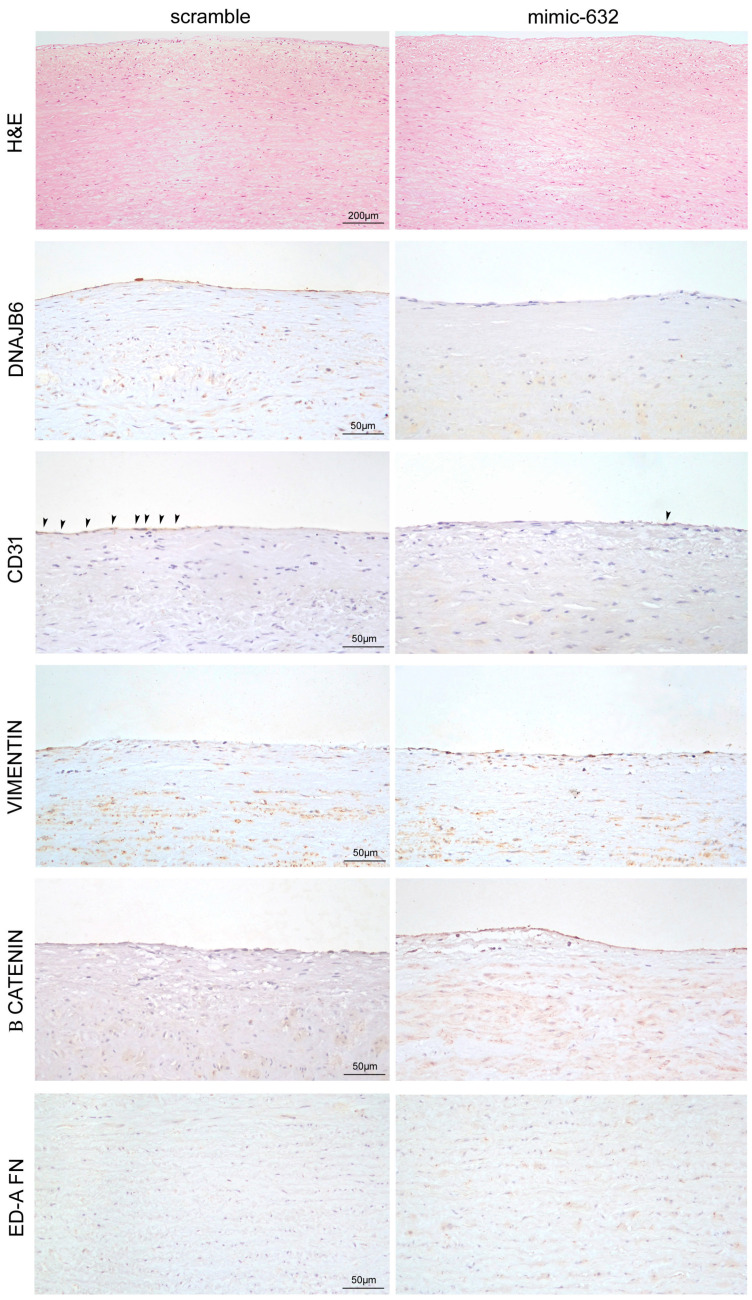
Microscopic features of the marked endothelial-to-mesenchymal transition and fibrosis induced by miR-632 overexpression. First row: representative images of hematoxylin and eosin-stained aorta tissue sections of non-MFS TAA transfected with mimic-632 or scramble for 1 week. Scale bar = 200 µm. Rows below: representative images of CD31, vimentin, β catenin, and ED-A FN immunostaining show a reduced percentage of CD31^+^ cells (arrowheads) and an increased percentage of vimentin and β catenin in the endothelium of miR-632 mimic-transfected non-MFS TAA. Regarding tunica media, representative images of β catenin and ED-A FN immunostainings also document an increased expression of those proteins in miR-632 mimic-transfected fragments. Scale bar = 50 µm. Experiments were performed in triplicate on one sample at a time for a total of six patients.

Those data were confirmed by the microscopic analysis of transfected non-MFS TAA tissue fragments after one week. In particular, we observed an increased endothelial and media degeneration, with an accumulation of basophilic material, in miR-632 mimic-transfected tissues (Figure 6H,E). Immunohistochemistry confirmed the strong downregulation of DNAJB6 and CD31, as well as vimentin, β catenin, and ED-A FN upregulation (Figure 6 and Table 1) in the endothelium and the tunica media of miR-632 mimic-transfected non-MFS TAA. 

### 2.6. TGF-β1 Treatment Upregulates miR-632 Inhibiting DNAJB6 Expression

**Figure 7:**
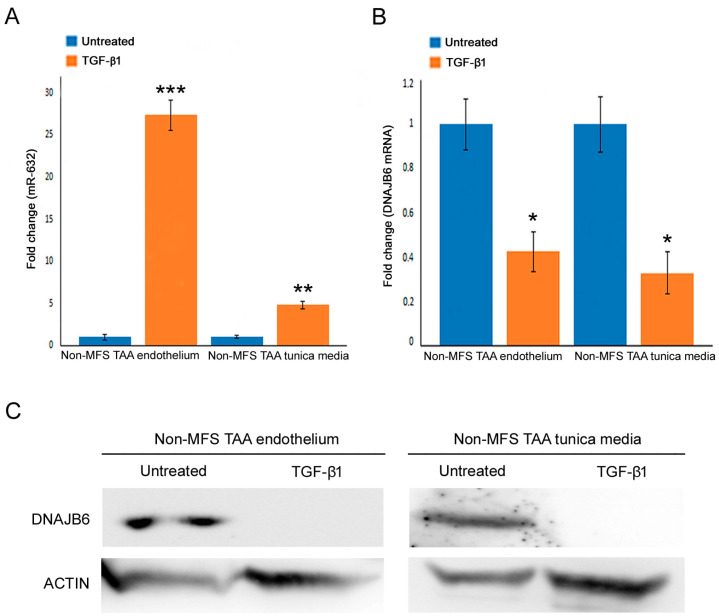
TGF-β1 treatment inhibits DNAJB6 by miR-632 upregulation. (**A**,**B**) Gene expression analysis shows a significant upregulation of miR-632 and DNAJB6 inhibition in the endothelium and the tunica media of TGF-β1-treated non-MFS TAA. The results are reported as the mean ± SEM. (**C**) Representative blots document the undetectable expression of DNAJB6 in TGF-β1-treated non-MFS TAA tissues. Experiments were performed in triplicate on one sample at a time for a total of six patients collected in two different pools (two for untreated and two for treated; three samples for each pool). Unpaired *t*-test: *, ** and *** indicate *p* < 0.05, *p* < 0.01 and *p* < 0.001, respectively; 1 < Cohen’s d < 11.

As mentioned, TGF-β signaling is directly implicated in the pathogenic mechanisms of MFS and indirectly involved in the Wnt/β catenin pathway [5]. Therefore, to verify a possible role of TGF-β in miR-632 signaling, non-MFS TAA tissue fragments were treated (or not treated) with 10ng/mL of TGF-β1. Expression analysis demonstrated the upregulation of miR-632 (Figure 7A; *p* < 0.001 and *p* < 0.01; d = 10.69 and d = 3.40), as well as a strong DNAJB6 downregulation in the endothelium and the tunica media of TGF-β1-treated non-MFS TAA (Figure 7B; *p* < 0.05; d = 1.39 and d = 4.70). 

### 2.7. TGF-β1 Treatment Favors Endothelial-to-Mesenchymal Transition and Smooth Muscle Cell Dedifferentiation

**Figure 8:**
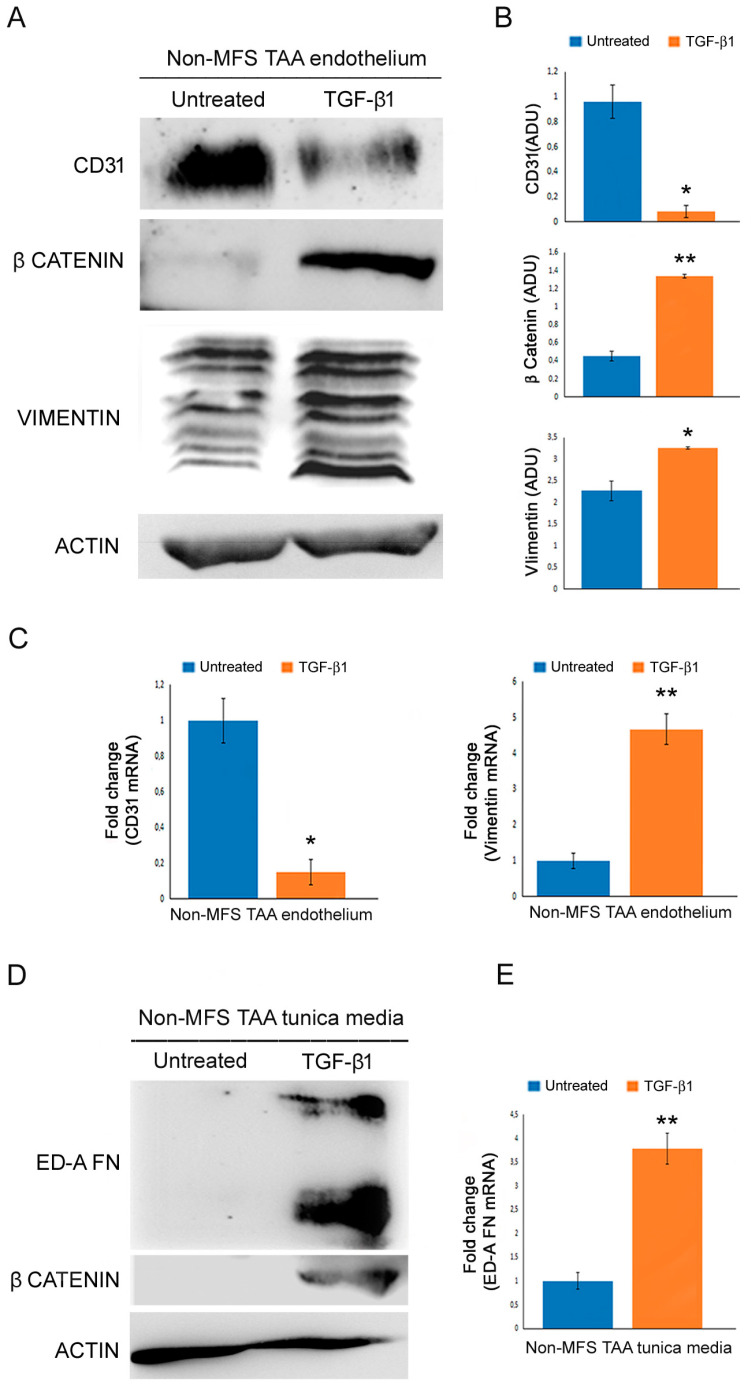
TGF-β1 treatment increases endothelial-to-mesenchymal transition and fibrosis. (**A**) Representative blots and (**B**) densitometric analysis show a reduced expression of CD31, as well as a high expression of β catenin and vimentin in the endothelium of TGF-β1-treated non-MFS TAA. (**C**) Gene expression analysis confirms the *CD31* downregulation and the upregulation of *VIMENTIN* in the endothelium of TGF-β1-treated non-MFS TAA. (**D**) Representative blots and (**E**) gene expression analysis document an increased expression of ED-A FN and β catenin in the endothelium of TGF-β1-treated non-MFS TAA. The results are reported as the mean ± SEM. Experiments were performed in triplicate on one sample at a time for a total of six patients collected in two different pools (two for untreated and two for treated; three samples each pool). Unpaired *t*-test: * and ** indicate *p* < 0.05 and *p* < 0.01, respectively; 1 < Cohens’d < 8.

We evaluated the effects of TGF-β1 stimulation on End-Mt and fibrosis in non-MFS TAA tissue fragments. We observed a strong downregulation of CD31 and vimentin upregulation in the endothelium of TGF-β1-treated non-MFS TAA (Figure 8A–C; *p* < 0.05 and *p* < 0.01, respectively; d = 4.71, d = 2.33, d = 5.20 and d = 7.36) and β catenin accumulation (Figure 8A,B; *p* < 0.01; d = 1.94). Regarding the tunica media, TGF-β1 treatment induced an accumulation of β catenin and the upregulation of ED-A FN (Figure 8D,E; *p* < 0.01; d = 4.15). 

**Figure 9:**
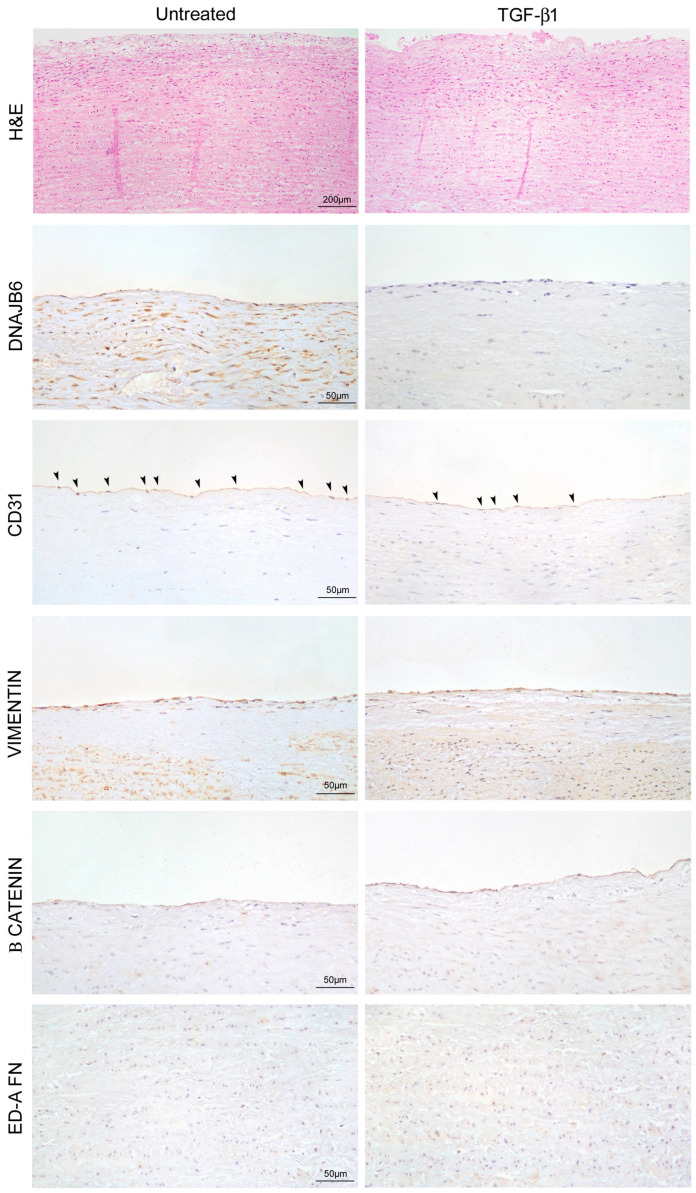
Microscopic features of the marked endothelial-to-mesenchymal transition and fibrosis induced by TGF-β1 treatment. First row: representative images of hematoxylin and eosin-stained aorta tissue sections of non-MFS TAA treated (or not treated) with TGF-β1 (10 ng/mL) for 1 week. Scale bar = 200 µm. Rows below: representative images of CD31, vimentin, β catenin, and ED-A FN immunostaining document the reduced percentage of CD31^+^ cells (arrowheads) and the increased percentage of vimentin and β catenin in the endothelium of TGF-β1-treated non-MFS TAA. Moreover, representative images of β catenin and ED-A FN immunostaining show the increased expression of those markers in the tunica media of TGF-β1-treated non-MFS TAA. Scale bar = 50 µm. Experiments were performed in triplicate on one sample at a time for a total of six patients.

Those data were also confirmed by histological analysis. In fact, we observed a greater degeneration of the endothelium and the tunica media, with an accumulation of basophilic material, in TGF-β1 treated-non-MFS TAA (Figure 9H,E). Immunohistochemical analysis confirmed the strong downregulation of DNAJB6 after TGF-β1 treatment, as well as the reduction in CD31 expression and the upregulation of vimentin, β catenin, and ED-A FN (Figure 9 and Table 2).

## 3. Discussion

**Figure 10:**
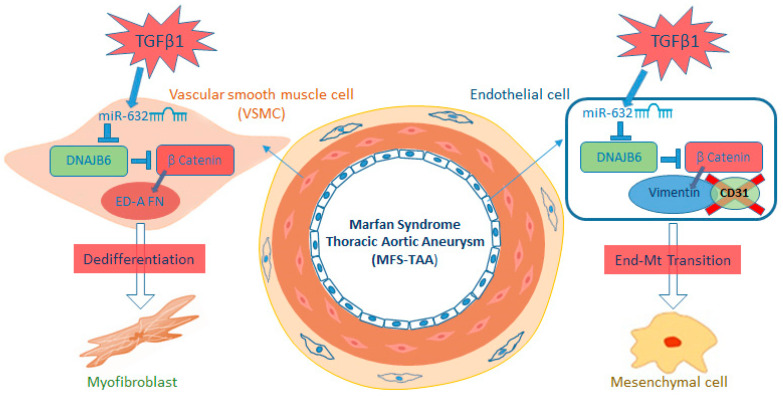
Schematic summary of the pathogenetic mechanisms induced by TGF-β1/miR-632 signaling in MFS TAA. MFS TAA is characterized by a hyperactivation of the TGF-β1 receptor that induces the upregulation of miR-632. The latter targets DNAJB6 and induces β catenin accumulation, favoring endothelial-to-mesenchymal transition and VSMC dedifferentiation (into myofibroblasts), with the consequent exacerbation of fibrosis.

Our present results documented that MFS TAA tissues overexpressed miR-632, compared with non-MFS TAA tissues. A previous study had established *DNAJB6* as a gene target for miR-632 in human breast cancer [16]. Consequently, we analyzed DNAJB6 expression, demonstrating its strong inhibition in both the endothelium and the tunica media of MFS TAA tissues. DNAJB6 is reported to be related to the Wnt/β catenin pathway, in particular by activating the DKK1 factor, which, in turn, blocks Wnt signaling, thereby inducing β catenin degradation and inhibiting the transcription of the genes involved in the Emt and, thus, metastatic progression [19,27]. Wnt/β catenin activation is also involved in myofibroblast dedifferentiation with consequent activation of the fibrotic process in several organs [26]. In the present study, we demonstrated that MFS TAA endothelial cells went through a severe “phenotypic switch” with the acquisition of mesenchymal features (End-Mt) through the hyperactivation of TGF-β signaling that, in turn, upregulated miR632, with the consequent downregulation of DNAJB6 and Wnt/β catenin activation (Figure 10). 

Those phenotypic changes lead to the impairment of the physiological functions of the endothelium, such as regulation of vascular permeability, control of homeostasis, and inflammation [28]. In addition, a more rapid transition to a mesenchymal phenotype of endothelium may affect smooth muscle cell contractility in the tunica media of the MFS aorta, which is already compromised due to the *FBN1* mutation [29]. 

The MFS tunica media showed the same deregulated pathways seen in the endothelium (Figure 10). In particular, we demonstrated that the hyperactivation of TGF-β signaling led to the smooth muscle cell dedifferentiation in myofibroblasts through the upregulation of miR632 and the consequent DNAJB6 downregulation and Wnt/β catenin activation. Those changes characterize the dedifferentiation of smooth muscle cells during the fibrotic process [25]. A correlation between genetic variants in TGF-β pathways and miRNA deregulation, including miR-632, has also been reported in colorectal carcinoma [30]. 

It should be noted that those processes (End-Mt and fibrosis) also characterize, in a less accentuated manner, non-MFS TAA [31]. In light of this, our study was not a comparison between a “normal” physiological condition and a pathological condition, but a comparison between a serious aortic wall degeneration (non-MFS TAA) and an even worse degeneration (MFS TAA). These findings derived from an ex vivo experimental aneurysm model, in which endothelium and the tunica media were analyzed in situ, keeping the characteristic 3D structure of aorta unaltered. This choice was made to preserve the natural microenvironment (cell–cell and cell–matrix interactions), the gene expression, and the phenotypic characteristics. In fact, it has been reported that the use of a bidimensional culture system alters the gene expression and the phenotypic features of endothelial and smooth muscle cells, making it difficult to study the endothelial-to-mesenchymal transition and smooth muscle cell dedifferentiation [32,33,34,35,36]. Moreover, as mentioned, the extracellular matrix (fibrillin) is fundamental for TGF-β activity, the interaction of which is altered in MFS TAA pathogenesis and progression. 

There are some limitations in the current work that should be considered. First, Marfan syndrome is a rare genetic disease; therefore, we were only able to collect and analyze a limited number of cases. Moreover, only small portions of excised aortas were analyzed. In addition, we focused our attention on DNAJB6, but it is well known that miRNAs have more than one gene target [37]. In fact, glycogen synthase kinase-3 beta (GS3KB), which is involved in the Wnt/β Catenin pathway, has also been reported to be an miR-632 target in the laryngeal carcinoma [38].

In conclusion, our findings deepen our understanding of some of the mechanisms implicated in the pathogenesis of MFS aortopathy. miR-632 and its target gene DNAJB6 could represent useful biomarkers for monitoring MFS disease progression and potential influencers in developing new specific therapeutic strategies.

## 4. Materials and Methods

### 4.1. Tissue Collection 

In this study, aortic tissue samples deriving from MFS patients (*n* = 30) and non-MFS TAA patients (*n* = 30) undergoing elective surgical procedures were collected between 2018 and 2022 in the Department of Cardiosurgery of Tor Vergata University of Rome. MFS diagnosis was made according to the clinical criteria defined by Ghent’s nosology [39]. Analysis of FBN1 mutations was performed on MFS patients by means of Ion S5 Next Generation Sequencing (Thermo Fisher Scientific, Waltham, MA, USA). All variant findings were then validated using Sanger Sequencing. Non-MFS patients were selected for non-genetic TAAs. Eligibility criteria were as follows: patients aged 20–50 years with TAAs, without coronary artery disease, endocarditis, aortic dissection, renal failure, liver disease, or tumor, and with a good ejection fraction. The mean ages of MFS patients and non-MFS patients were 30.5 years and 46 years, respectively. 

The diameter evaluation of ascending aorta was made both preoperatively and in the operating room by transthoracic echocardiography, and transesophageal echocardiography estimations were performed as follows: estimating the dimensions of the aortic annulus, the sinuses of Valsalva, and the proximal ascending aorta (above 2.5 cm of the sinotubular junction) in the parasternal long-axis view and evaluating the dimensions of the aortic arch from the suprasternal view (echocardiography-derived sizes were reported as internal diameter size [40]. Color Doppler was used to assess the presence and severity of aortic regurgitation and stenosis. The measurements of the aortic root and ascending aorta diameters were also carried out, using helical computed tomography image analysis techniques [40]. The surgical procedure was mostly the isolated ascending aorta replacement or the button Bentall operation, a modification of the original technique described by Kouchoukos et al. [41]. All operations were performed using crystalloid or hematic cardioplegia. The local ethics committee approved the study (protocol n. 179/18-01-Aorta-2018) and all patients signed the informed consent form.

### 4.2. Histochemical and Immunohistochemical Analysis 

Histochemical and immunohistochemical analyses were performed on excised aortic tissue samples in basal conditions and after treatments. Serial 4-µm thick paraffin sections from 10% neutral-buffered formalin-fixed aortic tissue samples were stained with Masson’s trichrome. The percentage of fibrotic area (collagen fiber accumulation) and the medial thickness were evaluated by using Image J software (1.50i, NIH, Bethesda, MD, USA), as previously reported [7]. Images were captured using a digital camera (DXM1200F, Nikon, Tokyo, Japan) connected to a light microscope (Eclipse E600, Nikon, Tokyo, Japan). For immunohistochemistry, sections were incubated with mouse monoclonal anti-DNAJB6 (1:100, Thermofisher, Waltham, MA, USA), anti-CD31 (1:50, Dako, Santa Clara, CA, USA), β catenin (1:50, Santa Cruz Biotechnology, Santa Cruz, CA, USA), anti-vimentin (1:200, Santa Cruz Biotechnology, Santa Cruz, CA, USA), and anti-ED-A FN (1:50, Santa Cruz Biotechnology), with positive and negative controls [42]. For the immunohistochemical evaluation, the percentage of positive cells/field (along the entire section, at 20× magnification) was calculated and reported as the mean ± SEM (standard error of mean) of the total fields.

### 4.3. Ex Vivo Transfection with Mimic for miR-632 of Aortic Tissue

Small fragments of fresh aortic tissue, derived from non-MFS TAA patients, were adhered to the bottom of a 24-well or 6-well multiwall (Supplemental Figure S1). Subsequently, ex vivo transfection with an miR-632 mimic (Merck KGaA, Darmstadt, Germany) was performed using Lipofectamin 2000 (Invitrogen, Thermo Fisher Scientific), according to the manufacturer’s protocols and previously reported studies [43,44]. First, transfection efficiency was assessed using the range 10–100nM of mimic and an incubation time between 12 h and 96 h. The maximum efficiency was obtained with 50 nm of mimic complexed to Lipofectamine (1:1), both diluted in OPTIMEM (Gibco-BRL, Monza, Italy). A non-specific sequence (scramble) was used as the control [43]. RNA and protein extraction was performed 24 h and 72 h after transfection, respectively. In some experiments, fragments were maintained in culture medium with the mimic for a week; then, the samples were collected and fixed in 10% neutral-buffered formalin for histological and immunohistochemical analyses. Experiments were performed in triplicate on one sample at a time for a total of six patients collected in two different pools (two for scramble and two for mimic-63; three samples for each pool).

### 4.4. TGF-β1 Ex Vivo Treatments of Aortic Tissue

Small fragments of fresh aortic tissue, deriving from non-MFS TAA, were adhered to the bottom of a 24- or 6-well multiwall (Supplemental Figure S1) and treated with 10 ng/mL of TGF-β1 (Sigma Aldrich, St. Louis, MO, USA) [45]. RNA and protein extraction was performed 24 and 72 h after transfection, respectively. In some experiments, fragments were maintained in culture medium with TGF-β1 for a week; then, samples were collected and fixed in 10% neutral-buffered formalin for histological and immunohistochemical analyses. Experiments were performed in triplicate on one sample at a time for a total of six patients collected in two different pools (two for untreated and two for treated; three samples for each pool).

### 4.5. Gene Expression Analysis

RNA extraction was performed on endothelium and tunica media of aortic tissue samples from MFS and non-MFS TAA. Before proceeding, aortic adventitia was removed and endothelium scraped. Total RNA and miRNA extraction was performed using the TRI Reagent^®^ (Sigma Aldrich, St. Louis, MI, USA) and the mirVana miRNA Isolation Kit (Thermo Fisher Scientific, Waltham, MA, USA) according to manufacturer’s protocols, as already reported [7]. 

Briefly, 700 ng of RNA were reverse-transcribed using the MystiCq microRNA cDNA Synthesis Mix kit (Sigma Aldrich, St. Louis, MI, USA) or the SuperScript III (Invitrogen, Thermo Fisher Scientific, Waltham, MA, USA). Real-time PCR was carried out by using SYBR Green (BioRad, Hercules, CA, USA). We used MystiCq microRNA Primer HSA-miR-632 (Merck KGaA, Darmstadt, Germany) and MystiCq Universal PCR Primer (Merck KGaA, Darmstadt, Germany) to analyze the expression of miR-632. In contrast, for gene expression analysis, cDNAs were amplified using specific primers (see Supplemental Table S1). HSA-miR-632 was normalized to the expression of *U6*, while the other genes were normalized to the expression of *GAPDH* (see Supplemental Table S1). Changes in target gene expression levels were calculated, using the comparative ΔΔCT method [46]. Fold change was considered significant for values > 2.0 and < 0.5. The analysis was performed in triplicate. For MFS TAA aorta analysis vs. non-MFS TAA aorta analysis, the assay was carried out on two different pooled samples (two for non-MFS and two for MFS; five samples for each pool), totaling ten MFS TAA patients and ten non-MFS TAA patients. 

### 4.6. Western Blot Analysis 

Total protein extraction was performed on the endothelium and the tunica media of aortic tissue samples from MFS TAA and non-MFS TAA. Before proceeding, aortic adventitia was removed and the endothelium was scraped. In order to analyze the expression of End-Mt markers, filters were incubated with specific primary antibodies: mouse monoclonal anti-DNAJB6 (1:300, Thermofisher, Waltham, MA, USA), anti-cd31 (1:200, Dako, Santa Clara, CA, USA), anti-Vimentin (1:200, Santa Cruz Biotechnology), and anti-β-catenin (1:200, Santa Cruz Biotechnology). For fibrosis markers, filters were incubated with specific primary antibodies: mouse monoclonal anti-DNAJB6 (1:300, Thermofisher), anti-β catenin (1:200, Santa Cruz Biotechnology), and anti-ED-A FN (1:200, Santa Cruz Biotechnology). Normalization was performed using mouse polyclonal anti-α-tubulin (1:700, Sigma Aldrich) or rabbit polyclonal anti-total actin (1:500, Sigma Aldrich). Detection and quantification were carried out as reported [47]. For MFS TAA vs. non-MFS TAA aorta analysis (two for non-MFS and two for MFS; five samples for each pool), totaling ten MFS TAA patients and ten non-MFS TAA patients. 

### 4.7. Statistical Analysis

The results were reported as the mean ± SEM (standard error of mean). In particular, we analyzed normally distributed and continuous variables between two independent experimental groups (non-MFS TAA vs. MFS-TAA, scramble vs. mimic-632, untreated vs. TGFβ1-treated); therefore, we calculated the differences between the means by using the unpaired *t*-test. Differences were considered statistically significant for a *p* value < 0.05. Moreover, we calculated the effect size through the Cohen’s d (indicated as “d”). All quantitative data sets presented here passed the normality tests. Statistical analyses were performed with SPSS software (IBM, SPSS Statistics, Chicago, IL, USA. version 23).

## Figures and Tables

**Table 1:** Immunohistochemical evaluation of End-Mt and fibrotic markers in transfected non-MFS TAA fragments (scramble or miR-632 mimic).

% of Positive Cells			
Marker	Scramble	Mimic MIR-632	*p* and d Value
CD31	49.2% ± 3.72 endothelial cells	9.50% ± 0.91 endothelial cells	*p* < 0,01; d = 5.97
VIMENTIN	23.13% ± 3.80 endothelial cells	69.45% ± 2.45 endothelial cells	*p* < 0.01; d = 6.23
β CATENIN	24.50% ± 4.50 endothelial cells	88.90% ± 3.0 endothelial cells	*p* < 0.01; d = 5.80
	5.25% ± 0.75 VSMCs	41.76% ± 3.76 VSMCs	*p* < 0.01; d = 5.57
ED-A FN	5.10% ± 0.51 VSMCs	32.30% ± 2.72 VSMCs	*p* < 0.01; d = 5.36

d = Cohen’s d value for effect size.

**Table 2:** Immunohistochemical evaluation of End-Mt and fibrotic markers on non-MFS aortic fragments treated with TGF-β1 (see Figure 9).

% of Positive Cells			
Marker	Untreated	TGF-β1	*p* and d Value
CD31	82.30% ± 1.60 endothelial cells	37.80 ± 2.80 endothelial cells	*p* < 0.01; d = 8.18
VIMENTIN	59.54% ± 3.54 endothelial cells	98.55% ± 1.45 endothelial cells	*p* < 0.01; d = 4.35
β CATENIN	39.39% ± 2.40 endothelial cells	86.74% ± 2.74 endothelial cells	*p* < 0.01; d = 7.34
	2.23% ± 0.27 VSMCs	19.01% ± 0.98 VSMCs	*p* < 0.01; d = 9.80
ED-A FN	1.82% ± 0.28 VSMCs	25.28% ± 1.72 VSMCs	*p* < 0.01; d = 7.80

d = Cohen’s d value for effect size.

## Data Availability

Data are contained within the article or in the Supplementary Material.

## Supplementary Materials

The supporting information can be downloaded at: https://www.mdpi.com/article/10.3390/ijms242015133/s1.
