# Supplementary material for: miR-632 Induces DNAJB6 Inhibition Stimulating Endothelial-to-Mesenchymal Transition and Fibrosis in Marfan Syndrome Aortopathy
Source: Int J Mol Sci. 2023 Oct 13;24(20):15133. doi: 10.3390/ijms242015133 (PMC10607153; doi:10.3390/ijms242015133)

Supplemental Figure S1. Treatment of TAA tissues for histological analysis.

Small fragments of fresh aortic tissue excised from surgical operated non-MFS TAA patients, after adventitia removal, were adhered to the bottom of a 24- or 6-well multiwell and maintained in culture medium with TGF-β1 or mimic-632 for one week. Then, samples were collected and fixed in 10% neutral-buffered formalin for histological and immunohistochemical analysis.

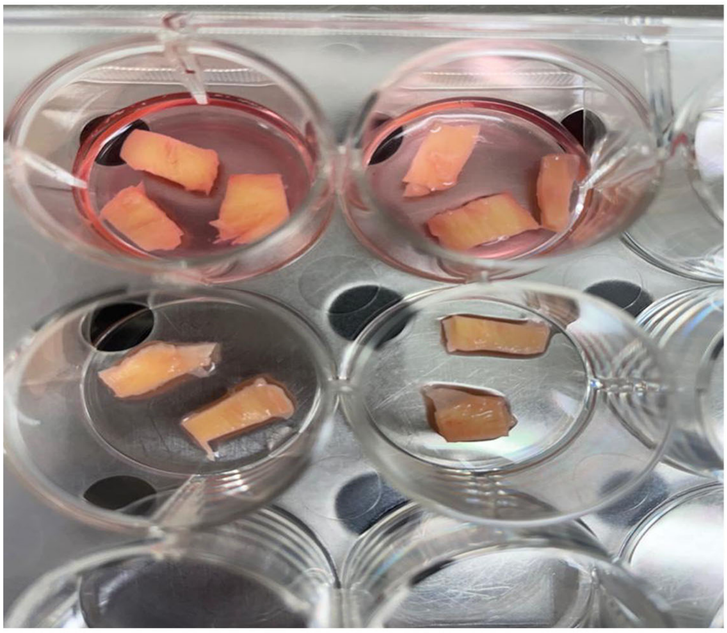

Supplement: Supplementary file 1 [file ijms-24-15133-s001.zip › Supplemental Figure S1.pdf]
