# Supplementary material for: miR-632 Induces DNAJB6 Inhibition Stimulating Endothelial-to-Mesenchymal Transition and Fibrosis in Marfan Syndrome Aortopathy
Source: Int J Mol Sci. 2023 Oct 13;24(20):15133. doi: 10.3390/ijms242015133 (PMC10607153; doi:10.3390/ijms242015133)
Supplement: Supplementary file 1 [file ijms-24-15133-s001.zip › Supplemental Table S1.pdf]

Supplemental Table S1. Sequences of primers used for Real-time PCR

PRIMER	SEQUENCES
**h-CD31**	Forward: 5’-GCCAGGTTGAGAAACTCTGC-3’
**h-DNAJB6**	Forward:5’-CATGCCTCACCCGAGGATATT-3’
**h-FN ED-A**	Forward: 5’-CCAGTCCACAGCTATTCCTG-3’
**h-VIMENTIN**	Forward: 5’-TCCAAGTTTGCTGACCTC-3’
**h-GAPDH**	Forward: 5’-ACGGATTTGGTCGTATTGG-3’
**hsa-miR-632**	MIRAP00623 (Merk Merck KGaA, Darmstadt, Germania)
**RNU6-1 (for miRNA)**	MIRCP00001 (Merk Merck KGaA)
